# Whole-blood expression of inflammasome- and glucocorticoid-related mRNAs correctly separates treatment-resistant depressed patients from drug-free and responsive patients in the BIODEP study

**DOI:** 10.1038/s41398-020-00874-7

**Published:** 2020-07-23

**Authors:** Annamaria Cattaneo, Clarissa Ferrari, Lorinda Turner, Nicole Mariani, Daniela Enache, Caitlin Hastings, Melisa Kose, Giulia Lombardo, Anna P. McLaughlin, Maria A. Nettis, Naghmeh Nikkheslat, Luca Sforzini, Courtney Worrell, Zuzanna Zajkowska, Nadia Cattane, Nicola Lopizzo, Monica Mazzelli, Linda Pointon, Philip J. Cowen, Jonathan Cavanagh, Neil A. Harrison, Peter de Boer, Declan Jones, Wayne C. Drevets, Valeria Mondelli, Edward T. Bullmore, the Neuroimmunology of Mood Disorders and Alzheimer’s Disease (NIMA) Consortium, Carmine M. Pariante

**Affiliations:** 1 Stress, Psychiatry and Immunology Laboratory & Perinatal Psychiatry, King’s College London, Institute of Psychiatry, Psychology and Neuroscience, Department of Psychological Medicine, Maurice Wohl Clinical Neuroscience Institute, King’s College London, SE5 9RT London, UK; 2 Biological Psychiatric Unit, IRCCS Istituto Centro San Giovanni di Dio Fatebenefratelli, 25125 Brescia, Italy; 3 Department of Pharmacological and Biomolecular Sciences, University of Milan, Milan, Italy; 4 Statistical Service, IRCCS Istituto Centro San Giovanni di Dio Fatebenefratelli, 25125 Brescia, Italy; 5 Department of Medicine, School of Clinical Medicine, University of Cambridge, Cambridge, CB2 0QQ UK; 6 Department of Psychiatry, School of Clinical Medicine, University of Cambridge, Cambridge, CB2 0SZ UK; 7 University of Oxford Department of Psychiatry, Warneford Hospital, Oxford, OX3 7JX UK; 8 Centre for Immunobiology, University of Glasgow and Sackler Institute of Psychobiological Research, Queen Elizabeth University Hospital, Glasgow, G51 4TF UK; 9 School of Medicine, School of Psychology, Cardiff University Brain Research Imaging Centre, Maindy Road, Cardiff, CF24 4HQ UK; 10 Neuroscience, Janssen Research & Development, Janssen Pharmaceutica NV, 2340 Beerse, Belgium; 11 Neuroscience External Innovation, Janssen Pharmaceuticals, J&J Innovation Centre, London, W1G 0BG UK; 12 Janssen Research & Development, Neuroscience Therapeutic Area, 3210 Merryfield Row, San Diego, CA 92121 USA

**Keywords:** Prognostic markers, Depression

## Abstract

The mRNA expression signatures associated with the ‘pro-inflammatory’ phenotype of depression, and the differential signatures associated with depression subtypes and the effects of antidepressants, are still unknown. We examined 130 depressed patients (58 treatment-resistant, 36 antidepressant-responsive and 36 currently untreated) and 40 healthy controls from the BIODEP study, and used whole-blood mRNA qPCR to measure the expression of 16 candidate mRNAs, some never measured before: interleukin (*IL)-1-beta*, *IL-6*, *TNF-alpha*, macrophage inhibiting factor (*MIF*), glucocorticoid receptor (*GR*), *SGK1*, *FKBP5*, the purinergic receptor *P2RX7*, *CCL2*, *CXCL12*, c-reactive protein (*CRP*), alpha-2-macroglobulin (*A2M*), acquaporin-4 (*AQP4*), *ISG15*, *STAT1* and *USP-18*. All genes but *AQP4*, *ISG15* and *USP-18* were differentially regulated. Treatment-resistant and drug-free depressed patients had both increased inflammasome activation (higher *P2RX7* and proinflammatory cytokines/chemokines mRNAs expression) and glucocorticoid resistance (lower *GR* and higher *FKBP5* mRNAs expression), while responsive patients had an intermediate phenotype with, additionally, lower *CXCL12*. Most interestingly, using binomial logistics models we found that a signature of six mRNAs (*P2RX7*, *IL-1-beta, IL-6*, *TNF-alpha, CXCL12* and *GR*) distinguished treatment-resistant from responsive patients, even after adjusting for other variables that were different between groups, such as a trait- and state-anxiety, history of childhood maltreatment and serum CRP. Future studies should replicate these findings in larger, longitudinal cohorts, and test whether this mRNA signature can identify patients that are more likely to respond to adjuvant strategies for treatment-resistant depression, including combinations with anti-inflammatory medications.

## Introduction

While there is overwhelming evidence of increased inflammation in depression^1–4^, the molecular signature underpinning this ‘pro-inflammatory’ phenotype is still unknown. A multitude of studies and meta-analyses show that patients with major depressive disorder (MDD) have, on average, increased serum levels of pro-inflammatory cytokines, like interleukin 1 beta (IL-1-beta), IL-6 and tumour necrosis factor alpha (TNF-alpha), and of the acute phase protein, C-reactive protein (CRP)^1,2,4,5^. Patients with ‘treatment resistant depression’ (TRD) are more likely to have increased inflammation^6,7^, as do patients with cardiovascular disorders, obesity, anxiety, and a history of childhood maltreatment^3,8–13^.

Whole blood mRNA expression analyses measure mRNAs coding for inflammatory genes and for genes operating upstream and downstream of these immune mechanisms, such as the glucocorticoid receptor (*GR*)^14^. We have been the first to demonstrate that drug-free depressed patients have increased mRNA expression *of IL-1-beta*, *IL-6* and *TNF-alpha*, together with reduced expression of the *GR* and increased expression of the FK506 binding protein 5 (*FKBP5*)^15^, which reduces GR function and promotes inflammation^16^. Together, these results suggest that inflammation in depression is potentially caused by escape of the immune system from the anti-inflammatory effects of glucocorticoid hormones (glucocorticoid resistance) as well as the pro-inflammatory effects of FKBP5^16^. Interestingly, we have also found that patients who do not respond to antidepressants have, before starting the antidepressant, higher levels of *IL-1-beta*, macrophage inhibiting factor (*MIF*) and *TNF-alpha* mRNAs, compared with antidepressant-responsive patients^15,17^. Separately, we have found increased mRNA expression of the GR-target gene, *SGK1*, in the blood of depressed patients, in human hippocampal cells treated with cortisol, and in the hippocampus of rats exposed to stress, thus indicating that mRNA in the human blood can reflect changes in the brain^18^.

Other blood mRNA studies on depressed patients have measured the whole genome, rather than focusing on a set of candidate genes, and have consistently found pro-inflammatory signatures. In one of the first such studies, Savitz et al.^19^ measured mRNA expression in peripheral blood mononuclear cells of depressed patients and identified differentially-expressed mRNAs that were linked to inflammatory pathway, such as nuclear factor kappa-B (NFkb), transforming growth factor beta (TGFb), and extracellular signal-regulated kinase (ERK). In the Netherlands Study of Depression and Anxiety (NESDA), Jansen et al. found an upregulation of IL-6- and natural killer cell-related related pathways^20^. Mellon et al. found over-expression of genes involved in Type I interferon responses, antimicrobial responses, and cytokine and chemokine signalling^21^, and we have recently found over-expression of genes specialised for innate immunity and myeloid cells^22^. Two studies using RNAseq have found differential regulation of type I interferon-related pathways^23,24^, with one study also showing enrichment for several other pathways involving immune function^23^. Finally, a very recent study has used genome-wide DNA methylation and gene expression analyses in patients prospectively-defined as responders and non-responders to an 8-week trial of escitalopram treatment^25^, and found two genes that exhibited increases in both DNA methylation and mRNA expression in non-responders: CHN2, which could affect hippocampal neurogenesis, and JAK2, which activates both innate and adaptive immunity.

In order to understand the specific molecular signatures associated with TRD vs. responsive depression, and their interaction with antidepressant treatment, in the present study we use whole blood mRNA quantitative polymerase chain reaction (qPCR) to measure the expression of 16 candidate mRNAs in 130 depressed patients (58 TRD, 36 antidepressant-responsive and 36 currently drug-free) and 40 healthy controls. We have recently published, in an overlapping sample, that only TRD patients have increased inflammation as measured as body mass index (BMI)-adjusted CRP^3^. Thus, here we hypothesise that TRD patients have the strongest mRNA-based evidence of inflammation and glucocorticoid resistance, as shown by higher expression of *IL-1-beta*, *IL-6*, *TNF-alpha* and *MIF*, together with lower *GR*, higher *FKBP5* and higher *SGK1* expression. Moreover, and *examining mRNA expression of genes hitherto unmeasured in psychiatric patients*, we hypothesise that this increased inflammation is associated with higher expression of the purinergic receptor, *P2RX7*, which mediates stress-induced activation of the inflammasome^26^; higher *CCL2* and lower *CXCL12* expression, as in the well-established animal model of ‘repeated social defeat’ (RSD) stress, characterised by increased inflammation and glucocorticoid resistance^27^; higher expression of *CRP* and of the other acute phase protein, alpha-2-macroglobulin (*A2M*)^4,28^; and higher expression of the interferon-responsive genes, acquaporin-4 (*AQP4*), *ISG15*, *STAT1* and *USP-18*, which we have recently shown to be elevated in the blood mRNA of patients with chronic viral hepatitis taking IFN-alpha^29^, an established model of inflammation-induced depression^30,31^, and to mediate the IFN-alpha-induced increase in neuronal apoptosis and decrease in neurogenesis^32^. Finally, to explore the clinical implications of these findings, we examined which genes would best classify depressed subjects in either TRD or antidepressant-responsive, even after adjusting for the effects of other clinical and immune variables, including serum CRP and white blood cells counts.

## Methods

### Study design and clinical measures

In total, 190 cases of MDD, meeting SCID-based DSM-5 criteria for a diagnosis for MDD^33^, and 54 healthy controls, were recruited in the non-interventional, case–control, Biomarkers of Depression (BIODEP) study^3^; 130 depressed patients and 40 healthy controls with available gene expression data are included in the present study. The cases were divided into three sub-groups based on current depressive symptom scores at the Hamilton Rating Scale for Depression (HAM-D), and current and previous drug treatment: (1) responsive patients *had no depressive symptoms* (HAM-D < 7) while currently on an antidepressant at standard therapeutic dose for at least 6 weeks; (2) drug-free *had depressive symptoms* (HAM-D > 17) and had not been medicated with any antidepressants for at least 6 weeks and (3) TRD patients *had depressive symptoms* (HAM-D > 13) while currently on an antidepressant at standard therapeutic dose for at least 6 weeks, plus they had at least one historical failure to a different antidepressant. Lifetime antidepressants use was measured using the antidepressant treatment response questionnaire (ATRQ)^34^, anxiety using the Spielberger State-Trait Anxiety Rating scale^35^ and exposure of stressors in childhood using the childhood trauma questionnaire (CTQ)^36^.

The study was part of the Wellcome Trust Consortium for Neuroimmunology of Mood Disorder and Alzheimer’s disease (NIMA), approved by the National Research Ethics Service East of England, Cambridge Central, UK (15/EE/0092). The study was conducted according to the Declaration of Helsinki, and all participants provided informed consent in writing.

### Clinical and sociodemographic features of the sample

Inclusion and exclusion criteria are presented in the Supplementary Material. The demographic and clinical characteristics of each group are summarised in Table 1. We had *n* = 58 TRD patients, *n* = 36 responsive patients, *n* = 36 drug-free patients and *n* = 40 healthy controls. Briefly, all the main within-group comparisons were similar to those already published in the larger sample^3^, and the groups did not differ significantly in age, gender distribution, educational level and BMI. As expected by design, each group differed significantly from the others on HAM-D total score (ANOVA, *F* = 683.6; d*f* = 3, 166; *P* < 0.001), with drug-free (HAM-D around 20) being more depressed than TRD (HAM-D around 18), and both being more depressed than responsive (HAM-D around 3) and controls (HAM-D less than 1). Moreover, both TRD and drug-free patients had higher state and trait anxiety compared with responsive and controls (ANOVA, *F* = 51.2 and 114.5, respectively; d*f* = 3, 166; *P* < 0.001). Finally, all patient groups had higher CTQ scores than controls, and both TRD and untreated patients had higher CTQ scores than responsive (generalised linear model (GLM), Wald chi-square = 106.6; d*f* = 1, 3; *P* < 0.001).

**Table 1:** Demographic, clinical and immune data.

	Mean [95% confidence interval]/*N* (%) in category				Group test		
	Healthy controls (Con) *N* = 40	Treatment-responders (Resp) *N* = 36	Drug-free (Free) *N* = 36	Treatment-resistant (TRD) *N* = 58	Statistic	*P* value	Post hoc #
Age, years [95% CI]	35.1 (32.7–37.5)	36.0 (33.2–38.7)	34.3 (31.8–36.9)	35.9 (34.0–37.8)	*F* = 0.43	0.73	
Gender, female, *N* [%]	26 (65.0%)	24 (66.7%)	23 (63.9%)	41 (70.7%)	Chi^2^ = 0.59	0.90	
Education level [below university yes/no %]	9/31 (22.5%/77.5%)	9/27 (25.0%/75.0%)	15/21 (41.7%/58.3%)	22/36 (37.9%/62.1%)	Chi^2^ = 14.6	0.26	
Relationship status [divorced, separated or single yes/no]	8/32 (20.0/80.0%)	13/23 (36.1/63.9%)	18/18 (50.0/50.0%)	30/28 (51.7/48.3%)	Chi^2^ = 21.6	0.01	
HAM-D total score [95% CI]	0.7 (0.3–1.0)	3.1 (2.5–3.8)	19.9 (19.0–20.9)	18.1 (17.3–18.9)	*F* = 683.6	<0.001	Each *vs* others
State anxiety [95% CI]	26.7 (24.7–28.7)	36.8 (33.2–40.4)	52.8 (49.0–56.6)	49.5 (46.1–52.8)	*F* = 51.19	<0.001	Con < others Resp vs. others
Trait anxiety [95% CI]	27.8 (26.2–29.5)	44.1 (40.4–47.8)	60.2 (56.8–63.9)	61.0 (58.2–63.9)	*F* = 114.5	<0.001	Con < others Resp vs. others
Number of failed antidepressants (lifetime) [95% CI]	0.0	0.83 (0.47–1.20)	0.89 (0.45–1.33)	1.74 (1.30–2.18)	*F* = 15.7	<0.001	Con < others TRD > others
Duration of exposure to antidepressants (lifetime) [95% CI]	0.0	20.7 (15.8–25.6)	18.9 (12.2–25.6)	24.6 (20.5–28.8)	*F*(2,101) = 1.31 (three groups)	0.27	
Total Score CTQ	40.1 (38.2–42.1)	47.6 (45.4–49.9)	54.1 (51.7–56.6)	53.4 (51.6–55.3)	Wald Chi^2^ = 106.6	<0.001	Con < others Resp vs. other
Smoking % current/past/never	12.8/25.6 /61.6	14.7/17.6 /67.7	11.4/20.0 /68.6	21.1/21.1 /57.8	Chi^2^ = 2.8	0.83	
Alcohol use % current/past/never	59.0/0.0 /41.0	54.3/14.3 /31.4	55.5/13.9 /30.6	63.8/3.4 /32.8	Chi^2^ = 9.9	0.13	
BMI, kg/m^2^	25.4 (23.8–27.0)	27.6 (25.6–29.7)	26.0 (24.6–27.3)	28.5 (26.3–30.7)	*F* = 2.35	0.073	
CRP, mg/L	1.1 (0.8–1.6)	2.2 (1.5–3.2)	2.9 (2.0–4.2)	5.0 (3.7–6.7)	Wald Chi^2^ = 40.49	<0.001	TRD > Con TRD > Resp Free > Cont
Total white cells	5.9 (5.5–6.4)	6.2 (5.5–6.9)	6.6 (6.1–7.2)	7.2 (6.6–7.7)	*F* = 4.09	0.008	TRD > Con
Lymphocytes absolute	1.9 (1.7–2.0)	1.9 (1.7–2.1)	1.9 (1.8–2.1)	2.1 (2.0–2.3)	*F* = 2.65	0.051	
Monocytes absolute	0.4 (0.35–0.44)	0.43 (0.37–0.49)	0.42 (0.38–0.47)	0.40 (0.37–0.44)	*F* = 0.46	0.710	
Neutrophils absolute	3.51 (3.14–3.89)	3.64 (3.15–4.41)	4.09 (3.60–4.57)	4.36 (3.92–4.80)	*F* = 3.30	0.022	TRD > Con
Basophils absolute	0.02 (0.02–0.03)	0.03 (0.02–0.03)	0.03 (0.02–0.03)	0.03 (0.02–0.03)	Wald Chi^2^ = 9.82	0.611	
Eosinophils absolute	0.15 (0.12–0.19)	0.19 (0.14–0.24)	0.18 (0.14–0.24)	0.23 (0.18–0.28)	Wald Chi^2^ = 6.22	0.101	

^#^Post hoc: ‘specific group category vs. others’ means that the specific group has mean score statistically different (larger or smaller) than the scores of others group categories;

‘one group > /< one group’ means that the first category group has score statistically larger/smaller than the second group.

Similar to the published larger sample^3^, the majority of TRD patients were currently taking selective serotonin reuptake inhibitors (72%), with smaller numbers exposed to noradrenergic and specific serotonergic reuptake inhibitors (14%), mirtazapine (9%), tricyclic antidepressants (4%) or bupropion (1%). Treatment-responsive patients were also predominantly taking selective serotonin reuptake inhibitors (69%), followed by noradrenergic and specific serotonergic reuptake inhibitors (22%) and mirtazapine (9%). Drug-free patients were *all currently not* on antidepressants for at least 6 weeks; however, *n* = 20 (55% of the group) had been on an antidepressant in the past, mostly (17 out of 20) on a selective serotonin reuptake inhibitor. As expected, the TRD group had more failed treatments than the other depressed groups (average of 1.7 vs. 0.8 in responders and 0.9 in drug free, ANOVA, d*f* = 3, 166; *P* < 0.001; see Table 1).

### Biomarkers

Venous blood was sampled from an antecubital vein between 08:00 and 10:00 h on the day of clinical assessment. Participants had fasted for 8 h, refrained from exercise for 72 h, and had been lying supine for 0.5 h prior to venepuncture. Whole blood (2.5 mL) was collected in PaxGene tubes at each recruitment site, and all PaxGene tubes were then kept at −80 °C and later transferred to a central site (Brescia) for RNA isolation and gene expression analyses. Isolation of total RNA was performed using the PAXgene blood miRNA kit according to the manufacturer’s protocol (PreAnalytiX, Hombrechtikon, CHE). RNA quantity and quality were assessed by evaluation of the A260/280 and A260/230 ratios using a Nanodrop spectrophotometer (NanoDrop Technologies, Delaware, USA) and by Agilent BioAnalyzer (Agilent Technologies); the RNA integrity number was above 8 for all sample. Samples were stored at −80 °C until processing.

Candidate gene expression analyses was performed using real-time PCR. For quality control, all samples were assayed in duplicate, and were randomised in different plates, also adding a calibrator, in order to control for possible differences in the efficiency of the Real Time reaction. Each target gene was normalised to the expression of three reference genes (glyceraldehyde 3-phosphate dehydrogenase, beta-actin, and beta-2-microglobulin). We used commercially-available Taqman primer and probes by using Taqman assays that are all available at the Thermofisher website (https://www.thermofisher.com/us/en/home/life-science/pcr/real-time-pcr/real-time-pcr-assays/taqman-gene-expression.html) on a 384-wells Real Time PCR System (Biorad); the assays had been already tested for efficiency by Thermo Fisher Scientific; catalogue numbers are available on request. The expression levels of each target gene were normalised to the geometric mean of all three reference genes, and the Pfaffl method was used to determine relative target gene expression of each gene in the patients’ groups compared with controls. The analyses were conducted by researchers who were blind to group allocation.

Methods for the immune assessments are described in the Supplementary Material.

### Statistical analyses

Socio demographic, clinical and immune measurements were compared among the four study groups by ANOVA, chi-square or GLM according to the statistical distribution of the variables (respectively, Gaussian, categorical and non-Gaussian). Group mean comparisons of the 16 genes were evaluated by ANOVA test followed by post hoc comparisons with Bonferroni correction. Correlations among the genes, as well as between genes and immune measures, were evaluated by Spearman’s rho coefficient. Binomial and multinomial logistic regression models were performed to detect the best predictors of the ‘study group’ outcome variable while adjusting for the effects of the other variables that were significantly different among the study groups in previous analyses. A stepwise-forward selection procedure was applied for the selection of the best (in terms of goodness of fit) predictors of the categorical ‘study group’ outcome, and predictive performances were evaluated by the Negelkerke pseudo-Rsquare goodness of fit index. Partial least square-discriminant analysis (PLS-DA) was conducted to define which genes contributed to discriminate between each study groups^37,38^; the contribution of each variable (gene) in the group discrimination was displayed by the loadings plots^39^. The data-reduction technique, principal component analysis (PCA), was used to derive, through the biplot, a graphical representation of the association between genes and subjects, labelled by study group (see Supplementary Material).

## Results

### TRD patients and drug-free depressed patients have the strongest signatures of inflammation and glucocorticoid resistance

TRD and drug-free depressed patients had increased levels of circulating serum CRP (see Table 1), as previously reported in the overlapping sample^3^. Specifically, CRP was higher in TRD patients compared with responsive and controls, and in drug-free patients compared with controls (GLM, Wald chi^2^ = 40.5; *P* < 0.001). Numerically, CRP was higher in TRD patients (average of 5 mg/L), followed by drug-free (2.9 mg/L), followed by responsive (2.2 mg/L), with controls averaging at around 1.1 mg/L. There were also significant differences in total white cell count (ANOVA, *F*_3,164_ = 4.09; *P* = 0.008) and absolute number of neutrophils (ANOVA, *F*_3,164_ = 3.3; *P* = 0.022): both were significantly higher in TRD patients compared with controls, and the gradient present for CRP (TRD > drug-free > responsive > controls) was present also for these measures.

Thirteen of the 16 genes were significantly different among the four groups (see Table 2, ANOVAs and post hoc comparisons with Bonferroni correction). In general, TRD and drug-free patients had similarly increased levels of inflammation-related genes: this applied to both the genes that had been measured before in depression (*IL1-beta*, *IL-6*, *TNF-alpha* and *MIF)* and those never measured before (*A2M, CRP*, *P2RX7*, *CCL2* and *STAT1*). Moreover, TRD and drug-free patients also showed similar evidence of glucocorticoid resistance (lower *GR* and higher *FKBP5* expression). Responsive patients had an intermediate phenotype with only some of these genes (*IL-6*, *MIF*, *TNF-alpha* and *A2M* as well as *FKBP5)* different from controls.

**Table 2:** Candidate gene expression data.

Genes	Mean expression levels [95% confidence interval]				Group test		
	Healthy controls (Con) *N* = 40	Treatment-responders (Resp) *N* = 36	Drug-free (Free) *N* = 36	Treatment-resistant (TRD) *N* = 58	Statistic	*P* value	Post hoc
*A2M*	1.02 [0.95–1.09]	1.28 [1.22–1.34]	1.24 [1.17–1.31]	1.23 [1.19–1.27]	*F* = 14.11	<0.001	TRD > Con Free > Con Resp > Con
*CRP*	1.03 [0.96–1.09]	1.13 [1.07–1.18]	1.18 [1.08–1.29]	1.18 [1.13–1.22]	*F* = 4.54	0.004	TRD > Con Free > Con
*IL-1beta*	1.07 [1.04–1.10]	1.16 [1.03–1.28]	1.22 [1.18–1.26]	1.32 [1.27–1.37]	*F* = 12.24	<0.001	TRD > Con TRD > Resp Free > Con
*IL-6*	1.06 [1.03–1.08]	1.32 [1.26–1.38]	1.28 [1.24–1.32]	1.23 [1.17–1.28]	*F* = 19.675	<0.001	TRD > Con Free > Con Resp > TRD Resp > Con
*MIF*	1.00 [0.96–1.05]	1.13 [1.07–1.20]	1.30 [1.24–1.37]	1.27 [1.23–1.30]	*F* = 29.62	<0.001	TRD > Con TRD > Resp Free > Con Free > Resp Resp > Con
*TNF-alpha*	1.06 [1.00–1.11]	1.24 [1.21–1.27]	1.30 [1.27–1.33]	1.32 [1.28–1.35]	*F* = 35.09	<0.001	TRD > Con TRD > Resp Free>Con Resp>Con
*P2RX7*	1.03 [0.95–1.12]	0.79 [0.74–0.84]	1.27 [1.13–1.40]	1.25 [1.20–1.30]	*F* = 29.69	<0.001	TRD > Con TRD > Resp Free > Con Free > Resp Con > Resp
*CCL2*	1.03 [0.99–1.06]	0.99 [0.94–1.05]	1.25 [1.20–1.29]	1.14 [1.11–1.17]	*F* = 27.485	<0.001	TRD > Con TRD > Resp Free > Con Free > Resp Free > TRD
*CXCL12*	1.06 [0.98–1.14]	0.93 [0.86–1.00]	1.03 [0.96–1.10]	1.08 [1.04–1.12]	*F* = 4.49	0.005	TRD > Resp Con > Resp
*AQP4*	1.03 [0.97–1.09]	1.03 [0.96–1.11]	1.03 [0.97–1.09]	1.08 [1.01–1.16]	*F* = 0.62	0.605	
*ISG15*	0.99 [0.91–1.06]	1.03 [0.95–1.12]	0.96 [0.88–1.04]	1.03 [0.95–1.10]	*F* = 0.64	0.59	
*STAT1*	1.06 [1.00–1.11]	1.08 [1.03–1.14]	1.23 [1.16–1.30]	1.19 [1.15–1.23]	*F* = 9.67	<0.001	TRD > Con TRD > Resp Free > Con Free > Resp
*USP18*	0.99 [0.91–1.07]	1.02 [0.93–1.10]	1.01 [0.95–1.08]	1.03 [0.98–1.09]	*F* = 0.245	0.865	
*FKBP5*	1.04 [0.97–1.10]	1.13 [1.08–1.18]	1.27 [1.23–1.30]	1.27 [1.25–1.29]	*F* = 30.31	<0.001	TRD > Con TRD > Resp Free > Con Free > Resp Resp > Con
*GR*	1.05 [1.02–1.08]	1.01 [0.97–1.05]	0.83 [0.80–0.87]	0.87 [0.84–0.90]	*F* = 40.28	<0.001	TRD < Con TRD < Resp Free < Con Free < Resp
*SGK1*	1.06 [1.03–1.09]	1.05 [1.02–1.08]	1.23 [1.20–1.26]	1.05 [1.02–1.08]	*F* = 32.34	<0.001	Free > Con Free > Resp Free > TRD

^#^Post hoc: ‘one group > /< one group’ means that the first category group has score statistically larger/smaller than the second group.

Contrary to our primary hypothesis that TRD patients would have the strongest evidence of inflammation and glucocorticoid resistance, none of the above genes were significantly higher in TRD compared with drug-free patients; indeed, *CCL2* was significantly higher in drug-free than in TRD patients (see Table 2). This suggests that TRD and drug-free patients came, at least in part, from phenotypically similar groups (see Discussion).

Interestingly, *SGK1* was significantly higher only in the drug-free group, while TRD and responsive patients had levels similar to controls. Thus, albeit elevated in depression as we hypothesised, *SGK1* levels were not linked with glucocorticoid resistance, since they were normal in TRD patients even if they had low *GR* mRNAs (see also correlation analyses below).

It is also of note that both *P2RX7* and *CXCL12* were *lower* in the responsive group compared with controls. For CXCL12, this confirms our hypothesis, based on the RSD animal model^27^, that this gene would be reduced in (at least some) patients with depression.

The three genes that were not differentially regulated were three of the four interferon-responsive genes, *AQP4*, *ISG15* and *USP-18*.

**Fig. 1:**
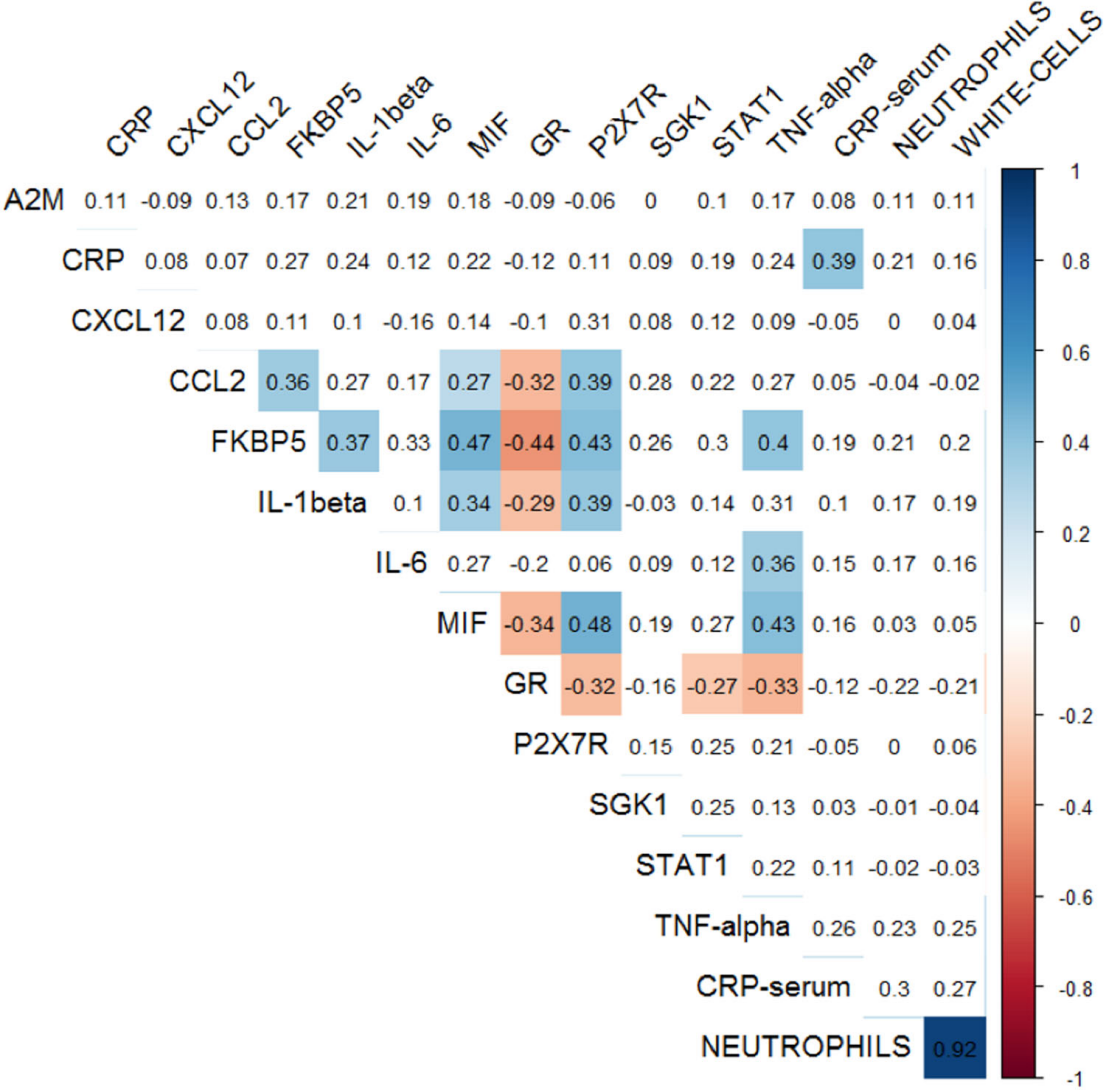
Correlations (Spearman’s rho) between significantly-different genes and immune measures. Coloured coefficients are statistically different from zero at level *P* < 0.05; red = negative correlations, blue = positive correlations.

The correlation matrix (Spearman’s rho) for 13 differentially expressed genes together with serum CRP and immune cell counts is presented in Fig. 1. There were significant, positive correlations between *P2RX7*, pro-inflammatory cytokines and *FKBP5* mRNAs, and significant negative correlations between all of these genes and *GR* mRNA. Moreover, white cell and neutrophil counts were (not-significantly) positively correlated with *FKBP5* (rh0 = 0.20/0.21) and negatively correlated with *GR* mRNA (rho = −0.21/−0.22). Together, these correlations indicate that, as hypothesised, the inflammasome/inflammatory gene over-expression and resulting immune activation are associated with glucocorticoid resistance and with FKBP5-mediated pro-inflammatory signalling. Interestingly, *GR* was negatively correlated with *FKBP5*, but neither was correlated with *SGK1*, confirming that SGK1 is not a marker of GR resistance. It is also of note that serum CRP (largely produced by the liver) was significantly, positively correlated with *CRP* mRNA (from the whole blood).

### Binomial logistic models show that a signature comprising P2RX7, IL-1-beta, IL-6, TNF-alpha, CXCL12 and GR, discriminates between TRD and responder patients over and above standard clinical and blood immune assessments

Binomial logistics models were performed applying the step-forward procedure, in order to examine the predicting performance of mRNA gene expression, clinical data and blood immune variables, in classifying depressed patients in the TRD or responders study group, while addressing the co-variance between the immune genes and adjusting for all the other clinical and immune variables (see Table 3).

**Table 3:** Binomial regression models output for detecting the best predictors of the binomial (two categories: Resp vs. TRD) study group variable.

Logistic models	Explanatory variables	Likelihood ratio test		Negelkerke’s pseudo-*R*^2^
		Chi^2^ (degree of freedom)	*P* value	
Mod. (i)	Trait-anxiety	23.9 (1)	<0.001	0.53
	State-anxiety	0.4 (1)	0.533	
	CRP	0.2 (1)	0.961	
	Neutrophils absolute	5.9 (1)	0.015	
	Total white cells	0.3 (1)	0.601	
	Total score CTQ	0.2 (1)	0.727	
Mod. (ii)	CXCL12	4.0 (1)	0.038	0.89
	CCL2	4.9 (1)	0.023	
	IL-1beta	3.8 (1)	0.048	
	IL-6	3.6 (1)	0.037	
	GR	18.4 (1)	<0.001	
	P2RX7	11.5 (1)	0.003	
	SGK1	2.2 (1)	0.125	
	TNF-alpha	3.7 (1)	0.042	
	FKBP5	4.5 (1)	0.004	
	A2M	2.1 (1)	0.076	
	MIF	6.1 (1)	0.018	
	STAT1	5.6 (1)	0.009	
	CRP	2.8 (1)	0.086	
Mod. (iii) ^#^	GR	5.7 (1)	0.017	0.90
	P2RX7	14.0 (1)	<0.001	
	TNF-alpha	4.1 (1)	0.040	
	Trait-anxiety	3.9 (1)	0.051	
	IL-6	4.2 (1)	0.042	
	CCL2	3.8 (1)	0.053	
	IL-1beta	6.6 (1)	0.010	
	CXCL12	5.7 (1)	0.031	
	Neutrophils absolute	1.2 (1)	0.277	
	FKBP5	2.4 (1)	0.124	
	MIF	2.5 (1)	0.113	
	STAT1	1.4 (1)	0.235	

^#^Explanatory variables of the model (iii) were standardised in order to take into account the different variable ranges.

Mod. (i) considering only (significantly different between group) clinical and blood immune variables; mod. (ii) considering only (significantly different between group) genes variables and mod. (iii) considering both genes and clinical-blood immune variables resulted remained significant in mod. (i) and (ii).

The first model included the six clinical and immune variables significantly different between the study groups (see Table 1): state anxiety, trait anxiety, total score CTQ, CRP, total white cells and neutrophils numbers. HAM-D and number of failed antidepressants were excluded as these were part of the decisional process leading to group allocation. Trait anxiety and neutrophils numbers were the only significant predictors, with a Nagelkerke’ pseudo-*R*-squared equal to 0.53.

The second model included the 13 significant genes from the univariate analyses (see ANOVA in Table 2). Ten genes were significant predictors (*P2RX7*, *IL-1b*, *IL-6*, *MIF*, *TNF-alpha*, *CCL2*, *CXCL12*, *GR*, *FKBP5* and *STAT1*), with a Nagelkerke’ pseudo-*R*-squared = 0.89.

Finally, the third model included the two significant variables from model 1 (trait anxiety and neutrophils number) and the ten significant genes from model 2. It resulted in six genes (*P2RX7*, *IL-1-beta*, *IL-6*, *TNF-alpha*, *CXCL12* and *GR*) remaining the only significant predictors, with a Nagelkerke’ pseudo-*R*-squared = 0.90. Thus, the expressions of these six genes remain significant predictors of the allocation of depressed patients to the TRD or responders group even after adjusting for the other clinical and immune variables, whose variability was fully captured by trait anxiety and neutrophils number, and with a larger predictive ability than the standard clinical and immune variables in Model 1 (Nagelkerke’ pseudo-*R*-squared = 0.90 vs. 0.53).

A second series of multinomial logistics models were performed to examine the predicting performance of gene expression, clinical data and blood immune variables, in classifying all study subjects in the four study groups, including drug–dree depressed patients and controls (see Supplementary Results and Supplementary Table 1). We found that a signature of five mRNAs (*P2RX7*, *IL-6*, *GR*, *SGK1* and *TNF-alpha*) together with trait anxiety significantly predicted the allocation of subjects to their study group.

### PLSDA show that P2RX7 best discriminates TRD patients vs. all other patients, while GR best discriminates responsive vs. all other depressed patients

**Fig. 2:**
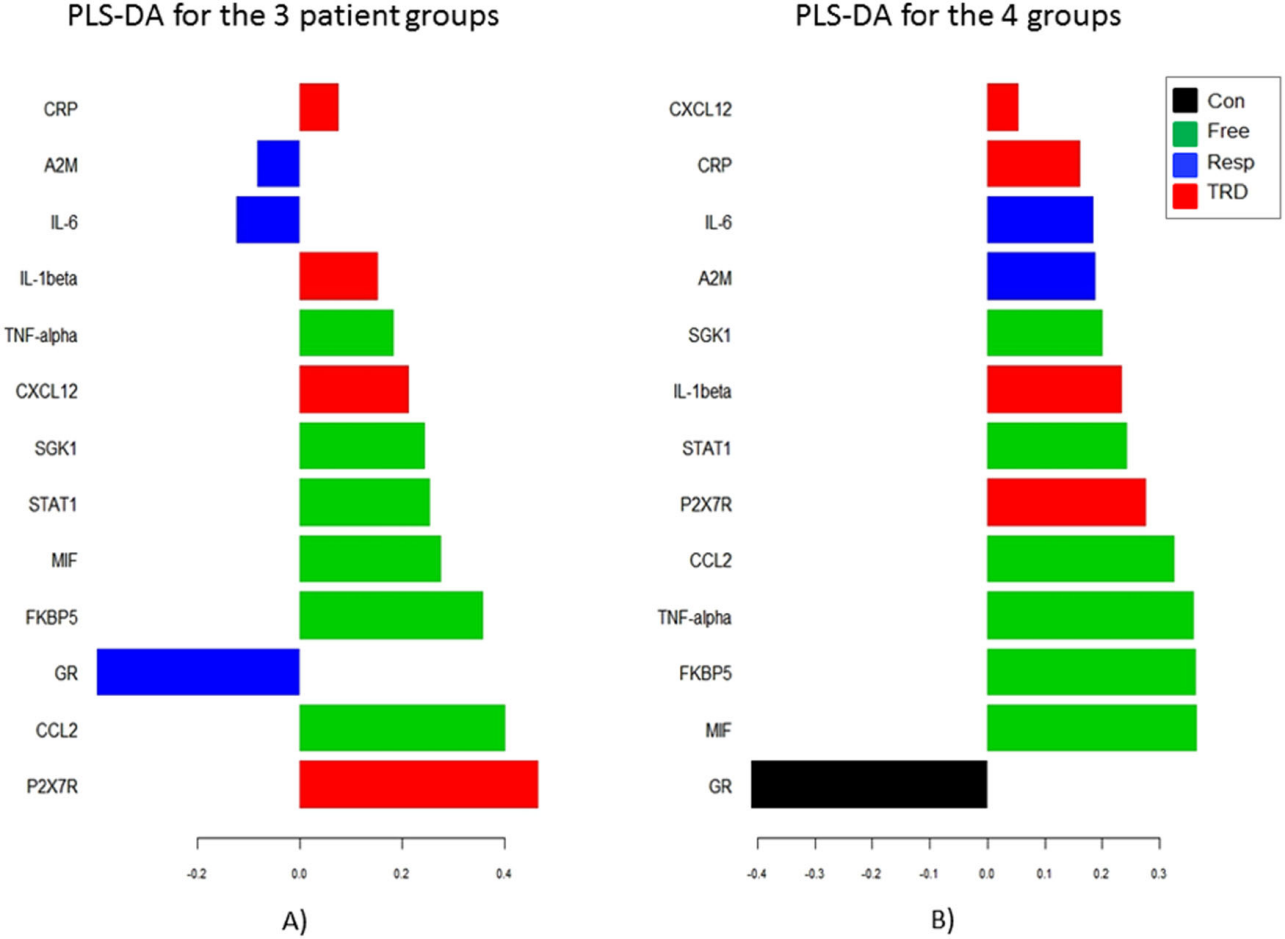
Partial least squares discriminant analysis outputs: loading plots. The partial least square discriminant analysis (PLSDA) was conducted to define which genes contribute to discriminate between each of the four groups. The plots depict the loadings of each gene: the larger the loading, the better the gene discriminates the study group from the others. Loadings summarise how the genes are related to each other as well as discriminate between the groups: all genes with positive loadings are positive correlated with each other and negatively correlated with genes with negative loadings; colours indicate the group for which the genes have a maximal median value. Panel A (on the three depressed groups only) shows that P2RX7, and, less, CXCL12 and IL-1-beta (all in red), best discriminate TRD vs. the other depressed groups; CCL2, and, less, FKBP5 and MIF (all in green), best discriminate drug-free vs the other depressed groups; and GR, and, less, IL-6 and A2M (all in blue), best discriminate responsive vs. the other depressed groups. Panel B (on the four groups) shows GR (in black) is the gene that best discriminates controls from all the other depressed groups.

The PLSDA is presented in Fig. 2. This was conducted to define which genes mainly contribute to discriminate between each of the four groups or between the three patient groups. Panel A (on the three depressed groups only) shows that: P2RX7, and, less, CXCL12 and IL-1-beta (all in red), best discriminate TRD vs. the other depressed groups; CCL2, and, less, FKBP5 and MIF (all in green), best discriminate drug-free vs. the other depressed groups; and GR, and, less, IL-6 and A2M (all in blue), best discriminate responsive vs. the other depressed groups. Panel B (on the four groups) shows GR (in black) as the gene that best discriminates controls from all the other depressed groups. It is worth noting that the discriminant performance of some genes overlaps on more than one patient group, as also indicated by the PCA of the 13 differentially expressed genes presented in Supplementary Material (Fig. S1).

## Discussion

In a study examining whole-blood mRNA expression of candidate genes in depressed patients characterised for their depressive symptoms and response to antidepressants, and testing both established and hitherto unmeasured mRNAs, we find evidence of inflammasome activation and glucocorticoid resistance in both drug-free depressed patients and antidepressant-treated TRD patients (less so in antidepressant-treated responsive patients). Moreover, a mRNAs signature of six genes (*P2RX7 and CXCL-12*, both measured for the first time in psychiatric patients, as well as *IL-1-beta, IL-6*, *TNF-alpha* and *GR*) is a significant predictor of allocation of depressed patients to the TRD or responder group in binomial logistics models, even after adjusting for other clinical variables that are different between groups, such as a history of childhood maltreatment and serum CRP.

Our data confirm our previous findings showing increased whole blood mRNA expression of *IL-6*, *MIF* and *TNF-alpha* in depressed patients vs. controls^15^, with higher levels of *IL-1-beta* and *MIF* predicting TRD when measured in drug free-depressed patients before starting an antidepressant treatment^15,17^. This consistency is particularly noticeable since the above-mentioned studies are clinical trials with a pre–post assessment^15,17^, and thus the biomarkers were measured before starting the antidepressants (at a time where patients were all drug-free and their response status was still unknown) and the response was measured prospectively. Admittedly, this was a much better design than the present study, which instead compares patients allocated to different groups based on a combination of current symptomatology and medication use as well historical treatment response. As shown in Table 1, these leads to groups that are different in a number of biological and clinical risk factors. All things considered, it is thus reassuring that we replicate both the increased *IL-6*, *MIF* and *TNF-alpha* in all our depressed groups vs. controls, as well as the increased *IL-1-beta, TNF-alpha* and *MIF* in TRD vs. responsive.

Meta-analyses of longitudinal studies have shown that antidepressant treatment (on average, for 6–12 weeks) is associated with decreases in serum or plasma cytokines, such as IL-6 and TNF-alpha, both in general^40^ and for SSRIs in particular^41^, with the most recent meta-analyses showing that TNF-alpha, but not IL-6, is differentially affected in responders only^42^. Data on longitudinal changes in mRNA expression are much more limited; for example, we published^15^ that 8-weeks of antidepressants (escitalopram or nortriptyline) decrease IL-6 mRNA, but this is driven by responders only, while TNF-alpha mRNA levels do not change. In the present study we find that levels of IL-6 and TNF-alpha mRNAs are higher in responders than controls, although with slightly different patterns, that is, responders have the highest IL-6 (higher even than TRD) while TNF-alpha is lower than in TRD patients. However, it is important to emphasise that it is difficult to compare the present study with all the others, because of the cross-sectional, rather than longitudinal, nature of our study: we simply do not know what the cytokines levels in these patients were before they started the antidepressants.

P2RX7 is a purinergic receptor that activates the NLR family pyrin domain containing 3 (NLRP3), a pattern-recognition receptor that precipitates the pro-inflammatory cascade^26,43^. P2RX7 is ubiquitously expressed in cells of the immune system^44^, but recent research has identified its expression also in neuronal cells, where it regulates the function of neurotransmitters relevant to depression^45^. In our study, *P2RX7* is not only associated with other markers of inflammation and with GR expression, as hypothesised, but it is also the strongest predictors of TRD in the PLSDA, and one of the predictive genes in the signature originated by the binomial and multinomial models. While one previous study found increased levels of NLRP3 in the monocytes of depressed patients^46^, the only evidence so far of a direct involvement of P2RX7 in depression comes from genetic studies associating a polymorphism in the gene with severity of depressive symptoms^45,47^.

We replicate here our previous findings showing reduced *GR* mRNA and higher *FKBP5* mRNA in depressed patients^15^. While increased FKBP5 expression is well known to induce glucocorticoid resistance^48,49^, new evidence indicates that FKBP5 can also directly promote inflammation by strengthening the interactions of NF-κB regulatory kinases^16^, and our findings showing that pro-inflammatory genes are positively correlated with *FKBP5* expression confirm these functional links. Indeed, the ultimate role of the reduced *GR* mRNA in our findings is difficult to define, as most clearly exemplified by the fact that responsive patients have *GR* levels indistinguishable from controls yet have increased *IL-6*, *MIF*, *TNF-alpha* and *A2M* levels. Moreover, recent data from the larger BIODEP sample show that only drug-free patients have increased cortisol levels^50^, but we show here that both drug-free and TRD have reduced GR mRNA. While the concept of reduced *GR* function and expression leading to ‘glucocorticoid resistance’ in depression has been extensively discussed before^51–55^, including for TRD patients^56–58^, the present study shows that reduced *GR* mRNA expression alone cannot fully explain the increased inflammation. Indeed the aforementioned study by Mellon et al.^21^ found upregulation of immune pathways in mononuclear cells from depressed patients in the absence of changes in GR function, and our own clinical meta-analysis on this topic has found only limited evidence linking ‘glucocorticoid resistance’ to inflammation^59^. Furthermore, it is important to emphasise here the additional confounding effects of antidepressant treatment. Previous studies have shown that antidepressants increase the expression and the function of the GR in experimental and clinical models^51,53,60,61^, and we have also found that GR mRNA levels are increased by antidepressants in the aforementioned longitudinal mRNA gene expression study, irrespective of response^15^. In the present study, we find that GR mRNA levels are ‘normal’ in responsive patients but lower in TRD, even if both groups have similar profiles of antidepressant treatment. In contrast, we find increased levels of the GR-target gene, *SGK1*, in drug-free depressed patients but not in antidepressant-treated (TRD and responsive) patients, and Frodl et al.^62^ also measured *SGK1* mRNA in depressed patients who were mostly on antidepressants and found no differences compared with controls. As mentioned above, the lack of longitudinal data in the present study makes it difficult to dissect the differential effects of antidepressant treatment vs. clinical improvement on mRNAs expression.

CCL2 and CXCL12 are chemokines involved in the RSD model of depression, characterised by increased inflammation and glucocorticoid resistance^27^. These mice show increased CCL2 in circulation and increased levels of the receptor for CCL2, C–C chemokine receptor type 2 (CCR2), in the brain, leading to monocyte recruitment to the brain and increased microglia activation. Consistently, we find increased *CCL2* mRNA expression in TRD and drug-free patients, and other studies found elevated serum CCL2 (also known as Monocyte chemoattractant protein 1, MCP-1) in depressed patients^63^. Interestingly, in the present study we find *lower* levels of *CCL2* in TRD patients than in drug-free patients (even if both are higher than in controls), and we have previously found, in a different sample, *lower* levels of serum CCL2 (MCP-1) in TRD vs. responsive patients^64^. Thus, it is possible that *lower* CCL2 in depression identifies a more severe, TRD group. Differently from CCL2, CXCL12 *inhibits* the trafficking of monocytes to the circulation, and in fact CXCL12 levels are *reduced* in the RSD model^55^. A recent meta-analysis did not find any studies measuring CXCL12 in depression^63^, but it is interesting that we find *reduced CXCL12* in responsive depressed patients in our study (and normal levels in the other depressed groups), showing some consistency with the RSD model.

Both *CRP* and *A2M* mRNAs are elevated in TRD and drug-free depressed patients in our study. There is an extensive literature showing elevated levels of serum (protein) CRP in depression, with more than 13,000 patients included in recent meta-analyses^2,4^ and evidence of increased CRP also in the cerebrospinal fluid^65^. Interestingly, while the liver is considered the most important source of CRP, *CRP* mRNA has been detected in macrophages from the lung^66^ and from atherosclerotic plaques^67^. Our study not only finds that *CRP* mRNA is expressed in circulating blood cells, but also that the whole-blood CRP mRNA is highly correlated with the levels of (liver-produced) serum CRP protein. A2M is another acute phase protein, like CRP, but there are only three studies looking at A2M serum levels in depression, with conflicting findings^68–70^. We have recently described higher *A2M* mRNA in both whole blood mRNA of adult humans exposed to early life trauma and the hippocampus of adult rats exposed to prenatal stress, and identified seven polymorphisms in the A2M gene that show significant gene × environment interactions with childhood stress in predicting depressive symptoms in adulthood^28^. Together, this evidence supports a role of A2M in depression, but further studies are needed.

Finally, we measure here the four interferon-responsive genes, acquaporin-4, ISG15, STAT1 and USP-18, which are elevated in the whole blood^29^ and in human neurones following IFN-alpha^32^. Only STAT1 is increased in the present study, in both drug-free and TRD patients, suggesting that the upregulation of the other three genes is only visible after pharmacological inflammation induced by IFN-alpha, or in brain tissue. Although this is the first study measuring STAT1 in the blood of depressed patients, the above-mentioned studies in the NESDA cohort^20^ and in non-responders to citalopram^25^ found an upregulation of, respectively, STAT3 and JAK2 mRNAs, and another study found STAT3 cell signalling alterations in depression^71^.

The study has two main limitations that must be discussed. Firstly, as mentioned above, this is not a clinical trial with pre–post measures of gene expression or longitudinal ascertainment of antidepressant resistance, and thus cross-sectional comparisons between groups are likely to be influenced by other clinical and sociodemographic variables that differ between groups. Of course, our analyses attempt to adjust for such group differences in the binomial/multinomial logistic regression models. Moreover, we had already measured the mRNA levels of seven of the 16 genes (*IL-1-beta*, *IL-6*, *TNF-alpha, MIF*, *GR*, *FKBP5* and *SGK1*) in drug-free depressed patients^15^ and in ‘prospectively-defined’ TRD patients^15,17,18^, and in the present paper we replicate all of these findings. Nevertheless, the cross-sectional design of the present study implies that, especially for the genes never measured before, the findings need to be replicated. The second important limitation is that the measurement of mRNA gene expression is in the whole blood rather than sorted immune cells. Of course, the ‘whole-blood’ approach has the advantages of speed and simplicity of blood collection and handling ‘at the bedside’, which is essential for the development of clinically useful biomarkers. However, we do not know which cells predominantly contributes to the mRNA findings, and furthermore we lack functional cellular data, for example, to measure inflammasome activation or glucocorticoid resistance. Thus, future studies should include an in-depth characterisation of immune cells-specific mRNA profiles as well as functional methodologies.

Notwithstanding these limitations, we believe that our paper is relevant to novel approaches for personalised psychiatry and novel targets for immune-related antidepressants therapies. We find that a combination of six genes (P2RX7, IL-1-beta, IL-6, TNF-alpha, CXCL-12 and GR) performs better than the routine clinical and immunological variables in identifying patients who are TRD or responsive to antidepressants. If replicated in larger, longitudinal samples, this signature might be helpful in identifying patients that should be fast-tracked into augmentation regimes—potentially a step toward overcoming the classic ‘trial and error’ approach in treating depression. In terms of novel targets, antagonists of P2RX7^72^, JAK^73^, CCR2^74^ and FKBP5^16^ are all novel antidepressant tools supported by our findings. Future studies will need to examine if these new treatments work, and whether responses to such new treatments can be improved by selecting patients with abnormal levels of relevant mRNAs^75^.

## Supplementary information

Supplementary Material

## References

[1] Haapakoski R., Mathieu J., Ebmeier K. P., Alenius H., Kivimäki M. Cumulative meta-analysis of interleukins 6 and 1β, tumour necrosis factor α and C-reactive protein in patients with major depressive disorder. *Brain Behav. Immun*. 10.1016/j.bbi.2015.06.001 (2015). 10.1016/j.bbi.2015.06.001 PMC4566946 26065825

[2] Osimo E. F., Baxter L. J., Lewis G., Jones P. B., Khandaker G. M. Prevalence of low-grade inflammation in depression: a systematic review and meta-analysis of CRP levels. *Psychol. Med*. 10.1017/S0033291719001454 (2019). 10.1017/S0033291719001454 PMC6712955 31258105

[3] Chamberlain S. R., et al. Treatment-resistant depression and peripheral C-reactive protein. *Br. J. Psychiatry*10.1192/bjp.2018.66 (2019). 10.1192/bjp.2018.66 PMC6124647 29764522

[4] Osimo E. F., et al. Inflammatory markers in depression: a meta-analysis of mean differences and variability in 5,166 patients and 5,083 controls. *Brain. Behav. Immun*. 10.1016/j.bbi.2020.02.010 (2020). 10.1016/j.bbi.2020.02.010 PMC7327519 32113908

[5] Dowlati Y., et al. A meta-analysis of cytokines in major depression. *Biol Psychiatry*10.1016/j.biopsych.2009.09.033 (2010). 10.1016/j.biopsych.2009.09.033 20015486

[6] Yang C., Wardenaar K. J., Bosker F. J., Li J., Schoevers R. A. Inflammatory markers and treatment outcome in treatment resistant depression: a systematic review. *J. Affect. Disord.* **257**, 640–649 (2019). 10.1016/j.jad.2019.07.045 31357161

[7] Strawbridge R., et al. Inflammation and clinical response to treatment in depression: a meta-analysis. *Eur. Neuropsychopharmacol*. 10.1016/j.euroneuro.2015.06.007 (2015). 10.1016/j.euroneuro.2015.06.007 26169573

[8] Nikkheslat N., et al. Insufficient glucocorticoid signaling and elevated inflammation in coronary heart disease patients with comorbid depression. *Brain Behav. Immun.*10.1016/j.bbi.2015.02.002 (2015). 10.1016/j.bbi.2015.02.002 25683698

[9] Baumeister D., Akhtar R., Ciufolini S., Pariante C. M., Mondelli V. Childhood trauma and adulthood inflammation: a meta-analysis of peripheral C-reactive protein, interleukin-6 and tumour necrosis factor-α. *Mol. Psychiatry*10.1038/mp.2015.67 (2016). 10.1038/mp.2015.67 PMC4564950 26033244

[10] Nettis M. A., et al. Metabolic-inflammatory status as predictor of clinical outcome at 1-year follow-up in patients with first episode psychosis. *Psychoneuroendocrinology* **99**, 145–153 (2019). 10.1016/j.psyneuen.2018.09.005 30243054

[11] Mondelli V., et al. Stress and inflammation reduce brain-derived neurotrophic factor expression in first-episode psychosis: a pathway to smaller hippocampal volume. *J. Clin. Psychiatry*10.4088/JCP.10m06745 (2011). 10.4088/JCP.10m06745 PMC4082665 21672499

[12] Hepgul N., et al. Childhood maltreatment is associated with increased body mass index and increased C-reactive protein levels in first-episode psychosis patients. *Psychol. Med*. 10.1017/S0033291711002947 (2012). 10.1017/S0033291711002947 PMC4081598 22260948

[13] Carvalho L. A., et al. Clomipramine in vitro reduces glucocorticoid receptor function in healthy subjects but not in patients with major depression. *Neuropsychopharmacology*10.1038/npp.2008.44 (2008). 10.1038/npp.2008.44 PMC3513411 18368033

[14] Hepgul N., Cattaneo A., Zunszain P. A., Pariante C. M. Depression pathogenesis and treatment: what can we learn from blood mRNA expression? *BMC Med*. 10.1186/1741-7015-11-28 (2013). 10.1186/1741-7015-11-28 PMC3606439 23384232

[15] Cattaneo A (2013). Candidate genes expression profile associated with antidepressants response in the GENDEP study: differentiating between baseline ‘predictors’ and longitudinal ‘targets’. Neuropsychopharmacology.

[16] Zannas A. S., et al. Epigenetic upregulation of FKBP5 by aging and stress contributes to NF-κB-driven inflammation and cardiovascular risk. *Proc. Natl Acad. Sci. USA*10.1073/pnas.1816847116 (2019). 10.1073/pnas.1816847116 PMC6561294 31113877

[17] Cattaneo A., et al. Absolute measurements of macrophage migration inhibitory factor and interleukin-1-β mRNA levels accurately predict treatment response in depressed patients. *Int. J. Neuropsychopharmacol.*10.1093/ijnp/pyw045 (2016). 10.1093/ijnp/pyw045 PMC5091822 27207917

[18] Anacker C., et al. Role for the kinase SGK1 in stress, depression, and glucocorticoid effects on hippocampal neurogenesis. *Proc. Natl Acad. Sci. USA*10.1073/pnas.1300886110 (2013). 10.1073/pnas.1300886110 PMC3666742 23650397

[19] Savitz J., et al. Inflammation and neurological disease-related genes are differentially expressed in depressed patients with mood disorders and correlate with morphometric and functional imaging abnormalities. *Brain Behav. Immun*. 10.1016/j.bbi.2012.10.007 (2013). 10.1016/j.bbi.2012.10.007 PMC3577998 23064081

[20] Jansen R (2016). Gene expression in major depressive disorder. Mol. Psychiatry.

[21] Mellon S. H., et al. Alterations in leukocyte transcriptional control pathway activity associated with major depressive disorder and antidepressant treatment. *Transl. Psychiatry*10.1038/tp.2016.79 (2016). 10.1038/tp.2016.79 PMC5070063 27219347

[22] Leday G. G. R., et al. Replicable and coupled changes in innate and adaptive immune gene expression in two case-control studies of blood microarrays in major depressive disorder. *Biol Psychiatry*10.1016/j.biopsych.2017.01.021 (2018). 10.1016/j.biopsych.2017.01.021 PMC5720346 28688579

[23] Le T. T., et al. Identification and replication of RNA-Seq gene network modules associated with depression severity. *Transl. Psychiatry*10.1038/s41398-018-0234-3 (2018). 10.1038/s41398-018-0234-3 PMC6125582 30185774

[24] Mostafavi S (2014). Type I interferon signaling genes in recurrent major depression: increased expression detected by whole-blood RNA sequencing. Mol. Psychiatry.

[25] Ju C., et al. Integrated genome-wide methylation and expression analyses reveal functional predictors of response to antidepressants. *Transl Psychiatry*10.1038/s41398-019-0589-0 (2019). 10.1038/s41398-019-0589-0 PMC6783543 31594917

[26] Bhattacharya A, Jones DN (2018). Emerging role of the P2X7-NLRP3-IL1β pathway in mood disorders. Psychoneuroendocrinology.

[27] Weber M. D., Godbout J. P., Sheridan J. F. Repeated social defeat, neuroinflammation, and behavior: monocytes carry the signal. *Neuropsychopharmacology*10.1038/npp.2016.102 (2017). 10.1038/npp.2016.102 PMC5143478 27319971

[28] Cattaneo A., et al. FoxO1, A2M, and TGF-β1: three novel genes predicting depression in gene X environment interactions are identified using cross-species and cross-tissues transcriptomic and miRNomic analyses. *Mol. Psychiatry*10.1038/s41380-017-0002-4 (2018). 10.1038/s41380-017-0002-4 PMC6283860 29302075

[29] Hepgul N., et al. Transcriptomics in interferon-α-treated patients identifies inflammation-, neuroplasticity- and oxidative stress-related signatures as predictors and correlates of depression. *Neuropsychopharmacology*10.1038/npp.2016.50 (2016). 10.1038/npp.2016.50 PMC4983179 27067128

[30] Capuron L., et al. Neurobehavioral effects of interferon-α in cancer patients: phenomenology and paroxetine responsiveness of symptom dimensions. *Neuropsychopharmacology*10.1016/S0893-133X(01)00407-9 (2002). 10.1016/S0893-133X(01)00407-9 11927189

[31] Capuron L., et al. Basal ganglia hypermetabolism and symptoms of fatigue during interferon-α therapy. *Neuropsychopharmacology*10.1038/sj.npp.1301362 (2007). 10.1038/sj.npp.1301362 17327884

[32] Borsini A., et al. Interferon-alpha reduces human hippocampal neurogenesis and increases apoptosis via activation of distinct STAT1-dependent mechanisms. *Int. J. Neuropsychopharmacol*. 10.1093/ijnp/pyx083 (2018). 10.1093/ijnp/pyx083 PMC5793815 29040650

[33] First M., Spitzer R., Gibbon, WilliamsJ. B. W. *Structured Clinical Interview for DSM-IV Axis Disorders—Patient Edition (SCID-I/P Version 2.0)*. (New York Biometrics Research Department, New York State Psychiatric Institute, 1996).

[34] Desseilles M., et al. Assessing the adequacy of past antidepressant trials: a clinician’s guide to the antidepressant treatment response questionnaire. *J. Clin. Psychiatry*10.4088/JCP.11ac07225 (2011). 10.4088/JCP.11ac07225 21899818

[35] Spielberger C. D., Gorsuch R. L., Lushene R. E. STAI manual for the state-trait anxiety inventory. Self-Evaluation Questionnaire. *MANUAL*10.1037/t06496-000 (1970).

[36] Bernstein D. P., et al. Initial reliability and validity of a new retrospective measure of child abuse and neglect. *Am. J. Psychiatry*10.1176/ajp.151.8.1132 (1994). 10.1176/ajp.151.8.1132 8037246

[37] Ferrari C, Macis A, Rossi R, Cameletti M (2019). Multivariate Statistical Techniques to Manage Multiple Data in Psychology.

[38] Barker M., Rayens W. Partial least squares for discrimination. *J. Chemometr*. 10.1002/cem.785 (2003).

[39] Brereton R. G., Lloyd G. R. Partial least squares discriminant analysis: taking the magic away. *J. Chemometr*. 10.1002/cem.2609 (2014).

[40] Köhler C. A., et al. Peripheral alterations in cytokine and chemokine levels after antidepressant drug treatment for major depressive disorder: systematic review and meta-analysis. *Mol. Neurobiol*. 10.1007/s12035-017-0632-1 (2018). 10.1007/s12035-017-0632-1 28612257

[41] Wang L., et al. Effects of SSRIs on peripheral inflammatory markers in patients with major depressive disorder: a systematic review and meta-analysis. *Brain Behav. Immun*. 10.1016/j.bbi.2019.02.021 (2019). 10.1016/j.bbi.2019.02.021 30797959

[42] Liu J. J., et al. Peripheral cytokine levels and response to antidepressant treatment in depression: a systematic review and meta-analysis. *Mol. Psychiatry*10.1038/s41380-019-0474-5 (2020). 10.1038/s41380-019-0474-5 31427752

[43] Farooq RK (2018). A P2X7 receptor antagonist reverses behavioural alterations, microglial activation and neuroendocrine dysregulation in an unpredictable chronic mild stress (UCMS) model of depression in mice. Psychoneuroendocrinology.

[44] Adinolfi E., et al. The P2X7 receptor: a main player in inflammation. *Biochem. Pharmacol*. 10.1016/j.bcp.2017.12.021 (2018). 10.1016/j.bcp.2017.12.021 29288626

[45] Ribeiro D. E., et al. P2X7 Receptor Signaling in Stress and Depression. *Int. J. Mol. Sci*. 10.3390/ijms20112778 (2019). 10.3390/ijms20112778 PMC6600521 31174279

[46] Alcocer-Gómez E., et al. NLRP3 inflammasome is activated in mononuclear blood cells from patients with major depressive disorder. *Brain Behav. Immun*. 10.1016/j.bbi.2013.10.017 (2014). 10.1016/j.bbi.2013.10.017 24513871

[47] Czamara D., Müller-Myhsok B., Lucae S. The P2RX7 polymorphism rs2230912 is associated with depression: a meta-analysis. *Prog. Neuropsychopharmacol. Biol. Psychiatry*10.1016/j.pnpbp.2017.11.003 (2018). 10.1016/j.pnpbp.2017.11.003 29122639

[48] Klengel T., et al. Allele-specific FKBP5 DNA demethylation mediates gene-childhood trauma interactions. *Nat. Neurosci.*10.1038/nn.3275 (2013). 10.1038/nn.3275 PMC4136922 23201972

[49] Klengel T, Binder EB (2015). Epigenetics of stress-related psychiatric disorders and gene × environment interactions. Neuron.

[50] Nikkheslat N (2020). Childhood trauma, HPA axis activity and antidepressant response in patients with depression. Brain Behav. Immun..

[51] Pariante C. M., Lightman S. L. The HPA axis in major depression: classical theories and new developments. *Trends Neurosci*. 10.1016/j.tins.2008.06.006 (2008). 10.1016/j.tins.2008.06.006 18675469

[52] Pariante C. M., Miller A. H. Glucocorticoid receptors in major depression: relevance to pathophysiology and treatment. *Biol. Psychiatry*10.1016/S0006-3223(00)01088-X (2001). 10.1016/s0006-3223(00)01088-x 11274650

[53] Pariante C. M. Why are depressed patients inflamed? A reflection on 20 years of research on depression, glucocorticoid resistance and inflammation. *Eur. Neuropsychopharmacol*. 10.1016/j.euroneuro.2017.04.001 (2017). 10.1016/j.euroneuro.2017.04.001 28479211

[54] Heim C., Newport D. J., Mletzko T., Miller A. H., Nemeroff C. B. The link between childhood trauma and depression: insights from HPA axis studies in humans. *Psychoneuroendocrinology*10.1016/j.psyneuen.2008.03.008 (2008). 10.1016/j.psyneuen.2008.03.008 18602762

[55] Niraula A, Wang Y, Godbout JP, Sheridan JF (2018). Corticosterone production during repeated social defeat causes monocyte mobilization from the bone marrow, glucocorticoid resistance, and neurovascular adhesion molecule expression. J. Neurosci..

[56] Juruena M. F., et al. prednisolone suppression test in depression: prospective study of the role of HPA axis dysfunction in treatment resistance. *Br. J. Psychiatry*10.1192/bjp.bp.108.050278 (2009). 10.1192/bjp.bp.108.050278 19336786

[57] Ising M., et al. The combined dexamethasone/CRH test as a potential surrogate marker in depression. *Prog. Neuropsychopharmacol. Biol. Psychiatry*10.1016/j.pnpbp.2005.03.014 (2005). 10.1016/j.pnpbp.2005.03.014 15950349

[58] Juruena M. F., et al. Different responses to dexamethasone and prednisolone in the same depressed patients. *Psychopharmacology*10.1007/s00213-006-0555-4 (2006). 10.1007/s00213-006-0555-4 17016711

[59] Perrin A. J., Horowitz M. A., Roelofs J., Zunszain P. A., Pariante C. M. Glucocorticoid resistance: is it a requisite for increased cytokine production in depression? A systematic review and meta-analysis. *Front. Psychiatry*10.3389/fpsyt.2019.00423 (2019). 10.3389/fpsyt.2019.00423 PMC6609575 31316402

[60] Pariante C. M., et al. Four days of citalopram increase suppression of cortisol secretion by prednisolone in healthy volunteers. *Psychopharmacology*10.1007/s00213-004-1925-4 (2004). 10.1007/s00213-004-1925-4 15179544

[61] Pariante C. M., Thomas S. A., Lovestone S., Makoff A., Kerwin R. W. Do antidepressants regulate how cortisol affects the brain? *Psychoneuroendocrinology*10.1016/j.psyneuen.2003.10.009 (2004). 10.1016/j.psyneuen.2003.10.009 14749091

[62] Frodl T (2012). Reduced expression of glucocorticoid-inducible genes GILZ and SGK-1: high IL-6 levels are associated with reduced hippocampal volumes in major depressive disorder. Transl. Psychiatry.

[63] Leighton S. P., et al. Chemokines in depression in health and in inflammatory illness: a systematic review and meta-analysis. *Mol. Psychiatry*10.1038/mp.2017.205 (2018). 10.1038/mp.2017.205 PMC5754468 29133955

[64] Carvalho L. A., et al. Lack of clinical therapeutic benefit of antidepressants is associated overall activation of the inflammatory system. *J. Affect. Disord*. 10.1016/j.jad.2012.10.036 (2013). 10.1016/j.jad.2012.10.036 23200297

[65] Felger J. C., et al. What does plasma CRP tell us about peripheral and central inflammation in depression? *Mol. Psychiatry*10.1038/s41380-018-0096-3 (2018). 10.1038/s41380-018-0096-3 PMC6291384 29895893

[66] Dong Q, Wright JR (1996). Expression of C-reactive protein by alveolar macrophages. J. Immunol..

[67] Kaplan M., et al. A significant correlation between C—reactive protein levels in blood monocytes derived macrophages versus content in carotid atherosclerotic lesions. *J. Inflamm*. 10.1186/1476-9255-11-7 (2014). 10.1186/1476-9255-11-7 PMC3944991 24588988

[68] SEIDEL A (1995). Cytokine production and serum proteins in depression. Scand. J. Immunol..

[69] Maes M (1992). Disturbances in acute phase plasma proteins during melancholia: additional evidence for the presence of an inflammatory process during that illness. Prog. Neuropsychopharmacol. Biol. Psychiatry.

[70] Jha MK (2017). Can C-reactive protein inform antidepressant medication selection in depressed outpatients? Findings from the CO-MED trial. Psychoneuroendocrinology.

[71] Lago S. G., et al. Exploring the neuropsychiatric spectrum using high-content functional analysis of single-cell signaling networks. *Mol. Psychiatry*10.1038/s41380-018-0123-4 (2018). 10.1038/s41380-018-0123-4 30038233

[72] Bhattacharya A. Recent advances in CNS P2X7 physiology and pharmacology: focus on neuropsychiatric disorders. *Front. Pharmacol*. 10.3389/fphar.2018.00030 (2018). 10.3389/fphar.2018.00030 PMC5799703 29449810

[73] Shariq AS (2018). Therapeutic potential of JAK/STAT pathway modulation in mood disorders. Rev. Neurosci..

[74] Noel M., et al. Phase 1b study of a small molecule antagonist of human chemokine (C-C motif) receptor 2 (PF-04136309) in combination with nab-paclitaxel/gemcitabine in first-line treatment of metastatic pancreatic ductal adenocarcinoma. *Invest. New Drugs*10.1007/s10637-019-00830-3 (2019). 10.1007/s10637-019-00830-3 PMC7211198 31297636

[75] Harris P. A., et al. Research electronic data capture (REDCap)—a metadata-driven methodology and workflow process for providing translational research informatics support. *J. Biomed. Inform*. 10.1016/j.jbi.2008.08.010 (2009). 10.1016/j.jbi.2008.08.010 PMC2700030 18929686

