# Correction: Whole-blood expression of inflammasome- and glucocorticoid-related mRNAs correctly separates treatment-resistant depressed patients from drug-free and responsive patients in the BIODEP study

**DOI:** 10.1038/s41398-020-01044-5

**Published:** 2020-10-19

**Authors:** Annamaria Cattaneo, Clarissa Ferrari, Lorinda Turner, Nicole Mariani, Daniela Enache, Caitlin Hastings, Melisa Kose, Giulia Lombardo, Anna P. McLaughlin, Maria A. Nettis, Naghmeh Nikkheslat, Luca Sforzini, Courtney Worrell, Zuzanna Zajkowska, Nadia Cattane, Nicola Lopizzo, Monica Mazzelli, Linda Pointon, Philip J. Cowen, Jonathan Cavanagh, Neil A. Harrison, Peter de Boer, Declan Jones, Wayne C. Drevets, Valeria Mondelli, Edward T. Bullmore, the Neuroimmunology of Mood Disorders and Alzheimer’s Disease (NIMA) Consortium, Carmine M. Pariante

**Affiliations:** 1 Biological Psychiatric Unit, IRCCS Istituto Centro San Giovanni di Dio Fatebenefratelli, 25125 Brescia, Italy; 2 Statistical Service, IRCCS Istituto Centro San Giovanni di Dio Fatebenefratelli, 25125 Brescia, Italy; 3 Department of Medicine, School of Clinical Medicine, University of Cambridge, Cambridge, CB2 0QQ UK; 4 Stress, Psychiatry and Immunology Laboratory & Perinatal Psychiatry, King’s College London, Institute of Psychiatry, Psychology and Neuroscience, Department of Psychological Medicine, Maurice Wohl Clinical Neuroscience Institute, King’s College London, SE5 9RT London, UK; 5 Department of Psychiatry, School of Clinical Medicine, University of Cambridge, Cambridge, CB2 0SZ UK; 6 University of Oxford Department of Psychiatry, Warneford Hospital, Oxford, OX3 7JX UK; 7 Centre for Immunobiology, University of Glasgow and Sackler Institute of Psychobiological Research, Queen Elizabeth University Hospital, Glasgow, G51 4TF UK; 8 School of Medicine, School of Psychology, Cardiff University Brain Research Imaging Centre, Maindy Road, Cardiff, CF24 4HQ UK; 9 Neuroscience, Janssen Research & Development, Janssen Pharmaceutica NV, 2340 Beerse, Belgium; 10 Neuroscience External Innovation, Janssen Pharmaceuticals, J&J Innovation Centre, London, W1G 0BG UK; 11 Janssen Research & Development, Neuroscience Therapeutic Area, 3210 Merryfield Row, San Diego, CA 92121 USA

Correction to: *Translational Psychiatry*

10.1038/s41398-020-00874-7

published online 23 July 2020

We have corrected this Article post-publication, because Dr. Cattaneo’s affiliation details were originally incorrect (she was affiliated with three institutions but is in fact only linked to one: Biological Psychiatry Unit, IRCCS Istituto Centro San Giovanni di Dio Fatebenefratelli, Brescia). These changes reflect in both the PDF and HTML versions of this Article.

