# Supplementary material for: Whole-blood expression of inflammasome- and glucocorticoid-related mRNAs correctly separates treatment-resistant depressed patients from drug-free and responsive patients in the BIODEP study
Source: Transl Psychiatry. 2020 Jul 23;10:232. doi: 10.1038/s41398-020-00874-7 (PMC7376244; doi:10.1038/s41398-020-00874-7)
Supplement: Supplementary file 1 — Supplementary Material [file 41398_2020_874_MOESM1_ESM.docx]

**WHOLE BLOOD EXPRESSION OF INFLAMMASOME- AND GLUCOCORTICOID-RELATED mRNAs CORRECTLY SEPARATES TREATMENT-RESISTANT DEPRESSED PATIENTS FROM DRUG-FREE AND RESPONSIVE PATIENTS IN THE BIODEP STUDY**

***SUPPLEMENTARY MATERIAL***

**NIMA MEMBERS DURING THE SAMPLE COLLECTION AND DATA ANALYSIS PERIOD FOR THE BIODEP STUDY**

Brighton & Sussex University Hospitals NHS Trust

Dominika Wlazly

Cambridgeshire & Peterborough NHS Foundation Trust

Amber Dickinson, Andy Foster, Clare Knight

Cardiff University

Claire Leckey, Paul Morgan, Angharad Morgan, Caroline O'Hagan, Samuel Touchard

GSK

Shahid Khan, Phil Murphy, Christine Parker, Jai Patel, Jill Richardson

Janssen

Paul Acton, Nigel Austin, Anindya Bhattacharya, Nick Carruthers, Peter de Boer, Wayne Drevets, John Isaac, Declan Jones, John Kemp, Hartmuth Kolb, Jeff Nye, Gayle Wittenberg

Kings College London

Gareth Barker, Anna Bogdanova, Heidi Byrom, Diana Cash, Annamaria Cattaneo, Daniela Enache, Tony Gee, Caitlin Hastings, Melisa Kose, Giulia Lombardo, Nicole Mariani, Anna McLaughlin, Valeria Mondelli, Maria Nettis, Naghmeh Nikkheslat, Carmine Pariante, Karen Randall, Julia Schubert, Luca Sforzini, Hannah Sheridan, Camilla Simmons, Nisha Singh, Federico Turkheimer, Vicky Van Loo, Mattia Veronese, Marta Vicente Rodriguez, Toby Wood, Courtney Worrell, Zuzanna Zajkowska

Lundbeck

Brian Campbell, Jan Egebjerg, Hans Eriksson, Francois Gastambide, Karen Husted Adams, Ross Jeggo, Thomas Moeller, Bob Nelson, Niels Plath, Christian Thomsen, Jan Torleif Pederson, Stevin Zorn

NHS Greater Glasgow and Clyde

Catherine Deith, Scott Farmer, John McClean, Andrew McPherson, Nagore Penandes, Paul Scouller, Murray Sutherland

Oxford Health NHS Foundation Trust

Mary Jane Attenburrow, Jithen Benjamin, Helen Jones, Fran Mada, Akintayo Oladejo, Katy Smith

Pfizer

Rita Balice-Gordon, Brendon Binneman, James Duerr, Terence Fullerton, Veeru Goli, Zoe Hughes, Justin Piro, Tarek Samad, Jonathan Sporn

Sussex Partnership NHS Foundation Trust

Liz Hoskins, Charmaine Kohn, Lauren Wilcock

University of Cambridge

Franklin Aigbirhio, Junaid Bhatti, Ed Bullmore, Sam Chamberlain, Marta Correia, Anna Crofts, Tim Fryer, Martin Graves, Alex Hatton, Manfred Kitzbichler, Mary-Ellen Lynall, Christina Maurice, Ciara O'Donnell, Linda Pointon, Peter St George Hyslop, Lorinda Turner, Petra Vertes, Barry Widmer, Guy Williams

University of Glasgow

Jonathan Cavanagh, Alison McColl, Robin Shaw

University of Groningen

Erik Boddeke

University of Oxford

Alison Baird, Stuart Clare, Phil Cowen, I-Shu (Dante) Huang, Sam Hurley, Simon Lovestone, Alejo Nevado-Holgado, Elena Ribe, Anviti Vyas, Laura Winchester

University of Southampton

Madeleine Cleal, Diego Gomez-Nicola, Renzo Mancuso, Hugh Perry

University of Sussex

Mara Cercignani, Charlotte Clarke, Alessandro Colasanti, Neil Harrison, Rosemary Murray

University of Texas

Jason O'Connor

University of Toronto

Howard Mount

**METHODS**

***Inclusion and exclusion criteria***

Patients were recruited from the UK National Health Service mental health, primary care services and from the general population by purposive advertising, while controls were recruited by purposive advertising. For all participants, the following inclusion criteria applied: age 25–50 years; able to give informed consent and understand English; able to fast for 8 h and abstain from strenuous exercise for 72 h prior to venous blood sampling. The following exclusion criteria applied: pregnancy or breast feeding, alcohol or substance use disorder in the preceding 12 months, participation in an investigational drug study within the preceding 12 months, lifetime history of any medical disorder or current use of any medication (e.g., statins, corticosteroids, antihistamines, anti-inflammatory medications) likely to compromise interpretation of immune measures, and lifetime histories of bipolar disorder or non-affective psychosis. Healthy controls had no current or past history of any major psychiatric disorder as defined by DSM-5, and no history of monoaminergic drug treatment for any indications.

***Standard immune assessments***

High-sensitivity CRP was measured using a Turbidimetry methods on Beckman Coulter AU analyzers, with rabbit anti-CRP-antibodies coated on latex particles. Absolute counts of total white blood cells, lymphocytes, neutrophils, monocytes, eosinophils and basophils were measured using Laser Particle Counting on a Coulter Hematology (LH750/DxH800).

**RESULTS**

Multinomial logistics models were performed to examine the predicting performance of gene expression, clinical data and blood immune variables, in classifying subjects in in the four study groups (see **Supplementary Table 1**), while addressing the co-variance between the immune genes and adjusting for all the other clinical and immune variables.

The first model included the six clinical and immune variables significantly different between the study groups (see Table 1): State Anxiety, Trait Anxiety, Total score CTQ, CRP, total white cells, and neutrophils numbers. HAM-D and number of failed antidepressants were excluded as these were part of the decisional process leading to group allocation. Only Trait Anxiety resulted as the strongest significant predictor, (i.e. the best predictor resulted by applying the step-forward procedure), with a Nagelkerke’ pseudo-R-squared equal to 0.67.

The second model included the 13 significant genes from ANOVA (see Table 2). The application of the step-forward procedure returned 8 genes as significant predictors of the group variable (*IL1b*, *IL-6*, *TNF-alpha*, *CCL2*, *CXCL12*, *GR*, *P2RX7*, *SGK1*) with a Nagelkerke’ pseudo-R-squared =0.97.

Finally, in the third model including Trait Anxiety and the 8 significant genes resulted from the previous models, five genes (*IL-6*, *GR*, *P2RX7*, *SGK1*, *TNF-alpha*) and Trait anxiety remain significant, with a Nagelkerke’ pseudo-R-squared =0.94. Thus, the expressions of these 5 genes remain significant predictors of the four study groups even after adjusting for the other clinical and immune variables, whose variability was fully captured by Trait Anxiety, and with a larger predictive ability than the standard clinical and immune variables in Model 1 (Nagelkerke’ pseudo-R-squared =0.94 vs. 0.67).

***The Principal Component Analysis for the set of 13 differentially expressed mRNAs shows that the partition between groups is driven by the contrast between higher pro-inflammatory mRNAs expression, mainly associated with TRD and drug-free patients, and higher GR expression, mainly associated with controls and responders***

We used the data-reduction technique Principal Component Analysis (PCA) to derive, through the biplot, a graphical representation of the association between genes and subjects, labelled by study group.

PCA for the set of 13 differentially expressed genes is presented in **Supplementary** **Figure 1**. As mentioned in the Methods, this was performed to derive a graphical representation of the association between genes and subjects labelled by study group. There was a partial overlap between TRD patients (in green) and drug-free patients (in blue), as well as a clear separation between these two groups and the control (in light pink) and responders (in violet). This partition between groups is consistent with the results of the univariate analyses (see main paper), and it is driven by the contrast between higher pro-inflammatory gene expression, mainly associated with TRD and drug-free patients, and higher *GR* expression, mainly associated with controls and responders.

**Supplementary Table 1**

**Multinomial logistic regression models output for detecting the best predictors of the multinomial (four categories) study group variable**

| **Logistic Models** | **Explanatory variables** | **Likelihood ratio test** | | **Negelkerke’s**  **Pseudo-R^2^** |
| --- | --- | --- | --- | --- |
|  |  | **Chi^2^ (degree of freedom)** | **P-value** |  |
| Mod. i) | Trait-Anxiety | 166.0 (3) | <0.001 | 0.67 |
|  | State-Anxiety | 5.5 (3) | 0.137 |  |
|  | CRP | 2.1 (3) | 0.554 |  |
|  | Neutrophils absolute | 6.8 (3) | 0.080 |  |
|  | Total White Cells | 2.5 (3) | 0.474 |  |
|  | Total Score CTQ | 0.4 (3) | 0.943 |  |
| Mod. ii) | CXCL12 | 9.0 (3) | 0.029 | 0.97 |
|  | CCL2 | 38.9 (3) | <0.001 |  |
|  | IL-1beta | 24.8 (3) | <0.001 |  |
|  | IL-6 | 35.5 (3) | <0.001 |  |
|  | GR | 68.4 (3) | <0.001 |  |
|  | P2RX7 | 61.5 (3) | <0.001 |  |
|  | SGK1 | 47.0 (3) | <0.001 |  |
|  | TNF-alpha | 16.0 (3) | 0.001 |  |
|  | FKBP5 | 3.3 (3) | 0.352 |  |
|  | A2M | 1.8 (3) | 0.611 |  |
|  | MIF | 1.1 (3) | 0.782 |  |
|  | STAT1 | 0.6 (3) | 0.898 |  |
|  | CRP | 0.1 (3) | 0.990 |  |
| Mod. iii) ^#^ | GR | 39.8 (3) | <0.001 | 0.94 |
|  | P2RX7 | 32.1 (3) | <0.001 |  |
|  | SGK1 | 54.9 (3) | <0.001 |  |
|  | TNF-alpha | 28.5 (3) | <0.001 |  |
|  | Trait-Anxiety | 65.6 (3) | <0.001 |  |
|  | IL-6 | 17.3 (3) | 0.001 |  |
|  | CCL2 | 6.6 (3) | 0.098 |  |
|  | IL-1beta | 6.1 (3) | 0.101 |  |
|  | CXCL12 | 4.4 (3) | 0.221 |  |

^#^ Explanatory variables of the model iii) were standardized in order to take into account the different variable ranges. Model i) considering only significant (see Table 1) clinical and blood immune variables; Model ii) considering only significant (see Table 2) genes variables; Model iii) considering both genes and clinical-blood immune variables resulted significant in Mod. i) and Mod ii).

**Supplementary Figure 1**

**Principal Component Analysis output: biplot representation**


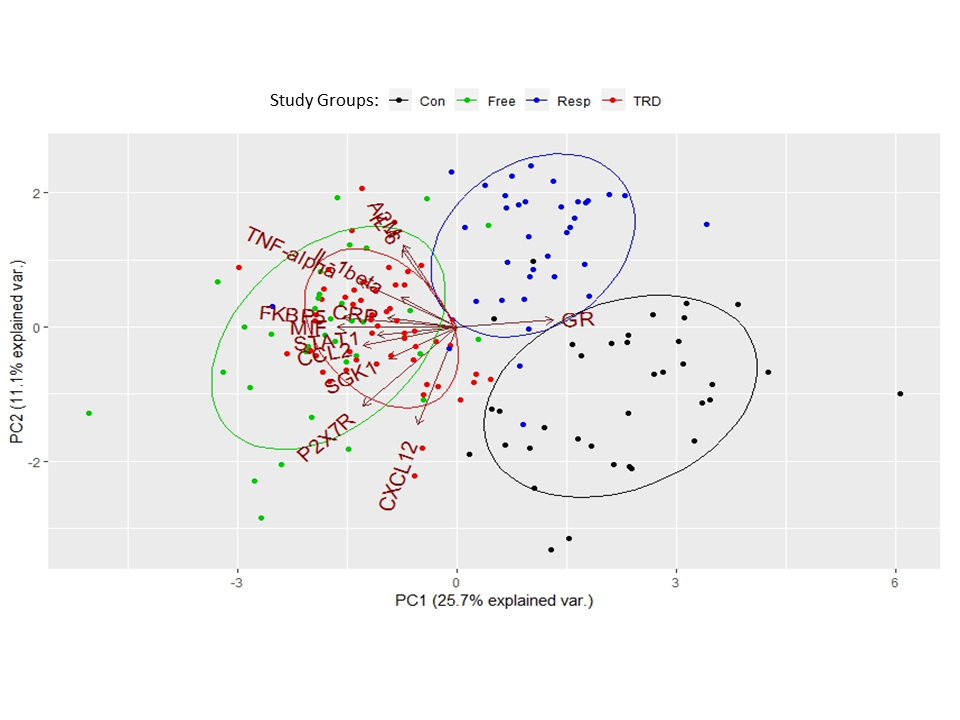


Principal component (PC) analysis for the set of 13 differentially expressed genes was performed to derive a graphical representation of the association between genes and subjects labelled by study group. There was a partial overlap between TRD patients (in red) and drug-free patients (in green), as well as a clear separation between these two groups and the control (in black) and responders (in blue). This partition between groups is consistent with the results of the univariate analyses (see Table 2) of the PLS-DA, and it is driven by the contrast between higher pro-inflammatory gene expression, mainly associated with TRD and drug-free patients, and higher *GR* expression, mainly associated with controls and responders. Arrows represent the variables (genes) and points represent the subjects labelled by study groups.
